# A Validated HPLC/MS Limit Test Method for a Potential Genotoxic Impurity in Cilostazol and its Quantification in the API and in the Commercially Available Drug Product

**DOI:** 10.3797/scipharm.1502-05

**Published:** 2015-03-26

**Authors:** Luigi Bray, Luca Monzani, Enrico Brunoldi, Pietro Allegrini

**Affiliations:** R&D Department, Dipharma Francis srl, Via Bissone 5, Baranzate (MI), Italy

**Keywords:** Cilostazol, Potential genotoxic impurity, HPLC/MS, Limit test method, Validation

## Abstract

Cilostazol is a selective inhibitor of type 3 phosphodiesterase. 5-(3-Chloropropyl)-1-cyclohexyl-1*H*-tetrazole, used as an intermediate in the synthesis of cilostazol, has a primary alkyl chloride group, a well-known alerting function for genotoxic activity. Upon request from a regulatory agency, a limit test in accordance with ICH Q2(R1) added with the accuracy of a recovery test of 5-(4-chlorobutyl)-1-cyclohexyl-1*H*-tetrazole in cilostazol was developed and validated. The application of the method highlighted the need to optimize the purification process to ensure levels of this potential genotoxic impurity in the final active pharmaceutical ingredient below the established limit. Also, the analytical method was suitable to determine the amount of the impurity in samples of the commercially available drug product, which showed the levels to be above the established threshold of toxicological concern (TTC).

## Introduction

Studying and monitoring the presence of impurities in Active Pharmaceutical Ingredient(s) (API(s)) is a central topic in drug production. In order to ensure proper quality levels in the manufactured products, consistent efforts are made to characterize, quantify, and monitor all the possible impurities present in the finished product. The limits of each impurity in the produced APIs are the subject of specific guidelines published by international agencies [1]. In the last several years, growing importance has been given to the quantification of potential genotoxic impurities [2–6], i.e. those which could cause DNA damage involving genetic mutations [7]. In order to make a preventive evaluation of the potential genotoxic activity of a given impurity, lists of alerting functions have been compiled on the basis of the structure of known genotoxic compounds and their mechanism of action [8, 9]: impurities bearing one or more alert functions have to be considered as potential genotoxic compounds if no toxicological data are available, and their limit has to be calculated according to specific guidelines [10, 11].

Cilostazol is a selective inhibitor of type 3 phosphodiesterase which is indicated for the improvement of the maximal and pain-free walking distance in patients with intermittent claudication [13]. Cilostazol can be produced according to the synthesis reported in Scheme 1 [14].

**Sch. 1:**
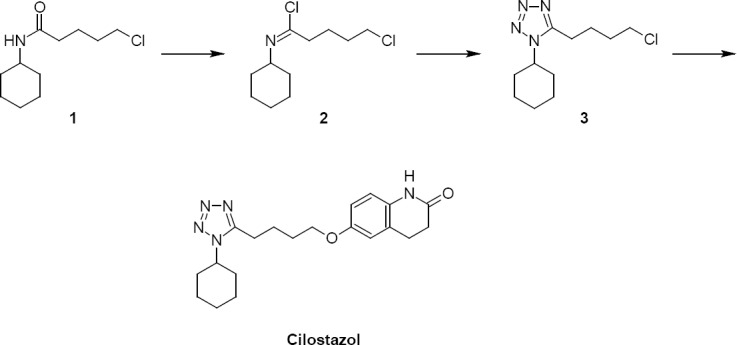
Synthetic process

As can be appreciated, the last intermediates are characterized by the presence of a primary alkyl chloride, a well-known alerting function for genotoxic activity [5, 8]. Thus, concerns arise about the presence of these compounds as residual impurities in production batches of cilostazol, as well as in the formulated drug. Since chloride **3** is the last intermediate of the synthesis, it is the one with the higher risk of carry-over into the final product. Indeed, samples of cilostazol produced according to the reported synthetic scheme were analyzed and resulted to contain compound **3** in variable amounts, ranging from 10 to 125 ppm. These results provided a clear indication that the carry-over of compound **3** in the finished API is not only a theoretical concern, but a real issue. Surprisingly, even if the majority of the industrial processes used for the production of cilostazol over the years present compound **3** as the last intermediate, to the best of our knowledge, no data are available in the literature about the potential genotoxicity of this compound. Moreover, no methods for the quantification of compound **3** are available in the literature. Also, the US Pharmacopoeia [12] does not provide any issue for the quantification of this alerting compound. For this reason, the problem of the carry-over of compound **3** had to be faced both from a regulatory and from an analytical point of view.

From a regulatory point of view, in order to assess the potential genotoxicity of **3**, a pure sample of compound **3** was subjected to the bacterial gene mutations test known as the Ames test [15]. However, the results of the Ames test did not bring forth any reliable conclusion regarding the genotoxic potential of compound **3**. Indeed, in the tests performed by an independent company, increases in the number of revertant colonies were found only for one out of the five bacterial strains tested, and only at the highest dosage level. These results precluded making a definite judgement about the mutagenicity of compound **3**, thus the study was considered inconclusive. For this reason, in the absence of reliable biological data, compound **3** had to be considered as a potential genotoxic impurity, for which limits can be calculated based on the Threshold of Toxicological Concern (TTC) approach [10]. Based on this approach, a limit of 7.5 ppm for potential genotoxic impurities should be calculated. However, a limit of 2.5 ppm for compound **3** was agreed upon with the involved regulatory agency. The choice of a lower limit took into account the possible carry-over of other potential genotoxic impurities related to the specific synthetic process, such as other intermediates or reagents used for the synthesis.

On the other hand, from an analytical point of view, the US Pharmacopoeia [12] describes the use of a high-performance liquid chromatography-ultraviolet (HPLC-UV) (254 nm) method which, unfortunately, is not sensitive enough to quantify **3** in the ppm range. Since the quantification of impurities in such a low amount is not routine in the conventional analysis of APIs, the development of a specific method for the quantification of chloride **3** in such a low amount was thus needed. The use of HPLC associated with mass spectrometry (MS) was chosen as the suitable analytical technique. Moreover, the use of single ion monitoring (SIM) as the detection method was expected to ensure sufficient sensitivity and specificity.

## Results and Discussion

### Method Development

In order to develop a suitable and convenient limit test method for compound 3 in cilostazol, HPLC associated with a UV detector, as described in the US Pharmacopoeia [12], was first tried due to its simple and widespread use in common quality control laboratories. However, as foreseen, this technique proved not sensitive enough for the quantification of 3 in the ppm range. The option of injecting a highly concentrated solution of cilostazol was precluded by its solubility limits in both the dissolution and elution phases. Thus, in order to get a higher sensitivity, the HPLC system with triple quadrupole and electrospray ionization (ESI) was chosen as the ionization technique. In the view of analyzing the complex mixtures such as those derived from formulated drugs, a method with high specificity was also desirable. The acquisition of the Total Ion Current (TIC) was considered not specific enough for the analysis of complex matrices. Thus, the Single Ion Monitoring (SIM) mode was chosen as the acquisition mode by monitoring the ion with m/z = 243 in positive ion mode, i.e. the ion generated by the simple protonation of 3.

### Method Validation

The validation performed was a limit test in accordance with ICH Q2(R1) with the addition of the accuracy of recovery [16] of 5-(4-chlorobutyl)-1-cyclohexyl-1*H*-tetrazole **3** in cilostazol. The parameters monitored in the validation study were specificity, accuracy, limit of detection (LOD), limit of quantification (LOQ), and the accuracy of recovery at the LOQ concentration.

**Fig. 1:**
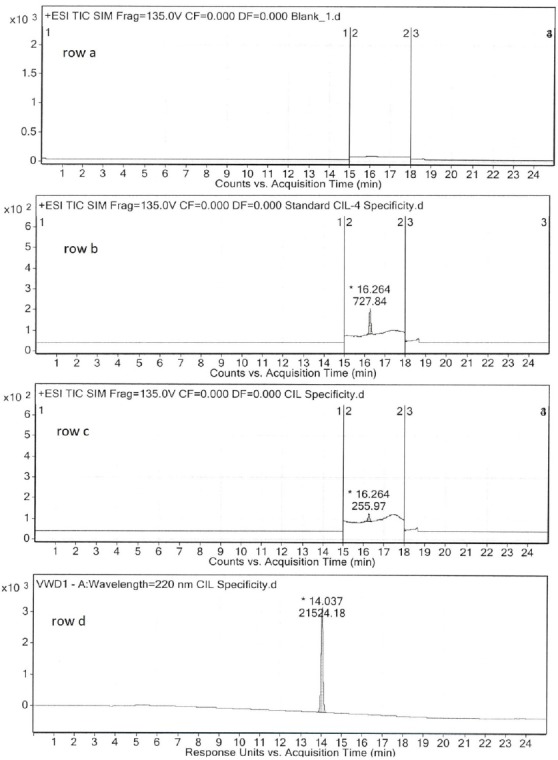
+ESI SIM chromatogram of a blank solution (row a); +ESI SIM chromatogram of a solution of compound **3** (row b); +ESI SIM chromatogram (row c); and HPLC-UV chromatogram (row d) of a solution of cilostazol

In order to demonstrate the specificity of the developed method, a blank, a solution of 5-(4-chlorobutyl)-1-cyclohexyl-1*H*-tetrazole **3** (25 ng/mL), and a solution of cilostazol (10 mg/mL) were prepared and analyzed. As a result, cilostazol eluted after 14.0 min while compound **3** eluted after 16.3 min. Thus, the SIM chromatogram was recorded from 15.0 to 18.0 min. No relevant interferences were observed around 16.3 min in the SIM chromatogram, the peak of compound **3** was the only one observed. Additionally, no relevant interferences were observed in the analysis of the blank sample (Figure 1).

In order to demonstrate the accuracy of the method, a solution of 5-(4-chlorobutyl)-1-cyclohexyl-1*H*-tetrazole **3** (25 ng/mL) was injected six times and the relative standard deviations (RDS%) were found to be 3.81%, which complies with the acceptance criteria of <5%. Moreover, the matrix solution was injected three times in order to evaluate the capability of the method’s recovery. The average area result was 186.1, with the RSD = 1.80%. With this value in hand, the capability of recovering compound **3** in the presence of cilostazol was verified through spiked recovery experiments: compound **3** was spiked into 10 mg/mL solutions of cilostazol (matrix) in triplicate at three levels, in the range of 50, 100, and 150% of the nominal concentration of 2.5 ppm (TTC-based limit). The average recovery resulted in the adequate range of 90–110%, with the RSD < 5% (Table 1).

**Tab. 1:**
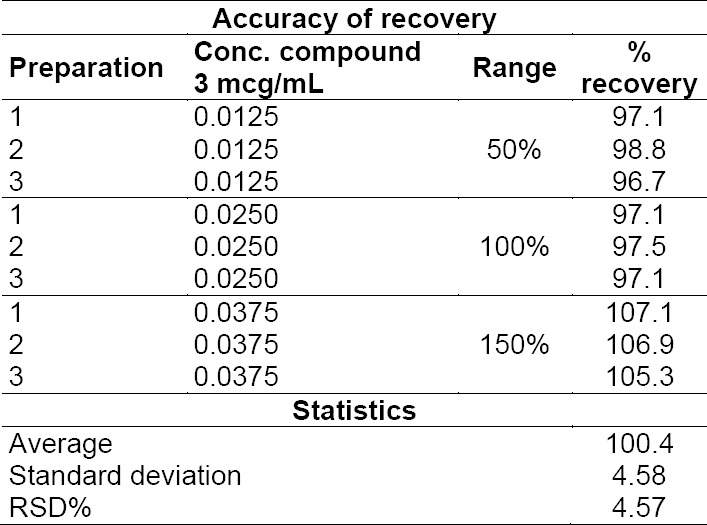
Results of the injections of the matrix solution spiked with 50%, 100%, and 150% of the nominal concentration of compound **3** (2.5 ppm) for the evaluation of the accuracy of recovery of the method

For the determination of the LOD and the LOQ concentration, the visual method of ICH Q2(R1) was adopted. A concentration of 0.102 ppm was selected as the LOD and a concentration of 0.34 ppm was selected as the LOQ. Both the LOD and the LOQ solutions were prepared and injected six times. The results obtained demonstrate that the LOD concentration is visible and that the LOQ concentration is quantifiable, with an RSD = 2.39 %.

Additionally, the accuracy of the recovery of compound **3** in cilostazol at the LOQ concentration was evaluated, by spiking 0.34 ppm of compound **3** into the matrix. The accuracy of recovery at the LOQ concentration was within the acceptance criteria of 90-110%, with the RSD < 5% (Table 2).

**Tab. 2:**
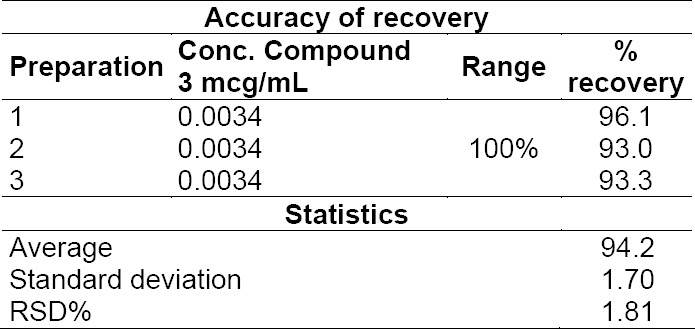
Results of the injections for the evaluation of the accuracy of the recovery of compound **3** at the LOQ concentration

The linearity of the method was also evaluated. The evaluation of the linearity of the method was not requested by ICH Q2(R1) for the validation of a limit test method, but was necessary for the quantification of compound **3** in the first batches of cilostazol analyzed and for the development of suitable purification conditions. Since the amount of compound **3** in cilostazol was initially unknown, compound **3** was injected at three different concentrations (2.54 ppm, 253.9 ppm, and 507.9 ppm) and the SIM peak area was plotted against the concentration. In the linearity correlation equation in the form Y = aX + b, where Y is the SIM peak area and X is the concentration of **3** in ppm, a resulted in 212.07 and b resulted in 572.52. The correlation coefficient (R2) = 0.9998 demonstrated the good linearity of the method. The validation of the limit test method in accordance with ICH Q2(R1) added with the accuracy of the recovery test was acknowledged by the involved regulatory agency.

### Sample Analysis

Since in preliminary analyses the levels of compound **3** in cilostazol resulted far above the set limit of 2.5 ppm, a specific optimization of the purification conditions was mandatory in order to bring the amount of compound **3** consistently below this value. After extensive efforts focused on the optimization of the purification process, 10 consecutive industrial batches of cilostazol were produced and analyzed in triplicate. As can be appreciated, in all cases the amount of compound **3** was found to be below the limit value of 2.5 ppm (Table 3).

**Tab. 3:**
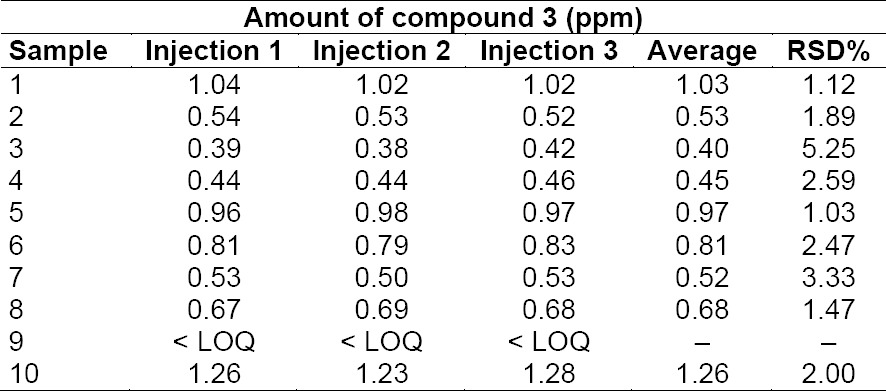
Amount of compound **3** in industrial batches of cilostazol produced via the optimised process

These results additionally support the fact that the carry-over of compound **3** in the final cilostazol is a real concern and they demonstrate that effective purification conditions are needed to ensure low amounts of this potential genotoxic impurity.

Subsequently, samples of commercially available cilostazol (tablets, 100 mg) were analyzed to check if the developed analytical method is suitable to detect compound **3** in the formulated product. No information was available about the synthetic process and the purification conditions used for the production of these formulations. Three tablets from the same batch were analyzed in triplicate. The results are reported in Table 4.

**Tab. 4:**
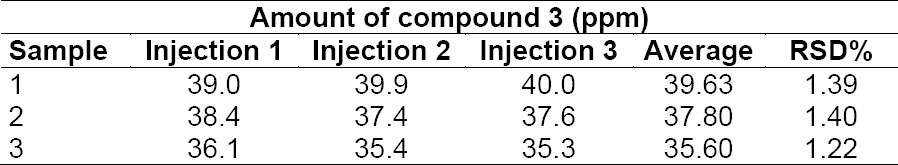
Amount of compound **3** in formulated cilostazol

As can be appreciated, compound **3** was detected in all of the samples in an amount much higher than the established threshold of 2.5 ppm. These results suggest that a high level of compound **3** in the API can directly reflect the high amounts in the formulated drug.

## Experimental

### Materials

HPLC grade acetonitrile and HPLC grade methanol were purchased from CARLO ERBA Reagents srl (Milan, Italy), formic acid was purchased from Sigma-Aldrich (Milan, Italy), water was obtained by using a Milli-Q system. 5-(4-Chlorobutyl)-1-cyclohexyl-1*H*-tetrazole **3** and cilostazol were supplied by Dipharma Francis srl (Mereto di Tomba, Italy).

### Chromatographic Separation

An Agilent Infinity 1200 Series high-performance liquid chromatography instrument was utilized for the chromatographic separation. It was equipped with a 1260 ALS autosampler, a 1290 thermostat, a 1260 Quant Pump VL, a 260 VWD detector, and a triple quadrupole 6420. The column was a Waters Sunfire 250 x 4.6 mm – 5 µm. One hundred percent acetonitrile was selected as solvent A, 0.1% formic acid in water (v/v) was selected as solvent B, and 100% methanol was selected as solvent C. The following reversed-phase gradient was used for a total of a 35 min sequence: start at (A:B:C 20/70/10) and increase to (A:B:C 70/20/10) in 15 min, hold at (A:B:C 70/20/10) for 10 min, lower back down to (A:B:C 20/70/10) in 1 min, and hold at (A:B:C 20/70/10) for 9 min. The column temperature and the flow rate were set, respectively, to 40°C and 0.8 mL/min, and the injection volume was set to 3 µL.

### Mass Spectrometry

The QQQ mass spectrometer was set on the single ion monitoring scan type (MS2 SIM) in positive ion mode. The monitored m/z value was 243±1. The voltage applied to the exit end of the capillary was 135 V with a collision cell accelerator voltage (CAV) of 7 V. The selected source parameters were: gas temperature 300°C, gas flow 13 L/min, nebulizer 20 psi, and capillarity 4900 V+.

### Standard Solutions and Sample Preparation

The sample was dissolved in a dissolution phase (DP) consisting of acetonitrile:water 80/20. For the preparation of a standard solution of compound **3** (2.5 ppm), 25 mg of compound **3** were weighed in a 100-mL volumetric flask and brought to volume with DP. One mL of the resulting solution (0.25 mg/mL) was diluted to 100 mL with DP. 1 mL of the resulting solution (0.0025 mg/mL) was diluted again to 100 mL with DP. For the preparation of the test solutions, 100 mg of cilostazol were weighed in a 10-mL volumetric flask and brought to volume with DP.

## Conclusion

5-(4-Chlorobutyl)-1-cyclohexyl-1*H*-tetrazole **3** was identified as a potential genotoxic impurity in cilostazol due to the presence of a well-known alerting function, i.e. a primary alkyl chloride. Since the Ames test did not bring forth any reliable conclusion regarding the genotoxic potential of compound **3**, it had to be considered as a potential genotoxic impurity. The validation of the HPLC/MS limit test method in accordance with ICH Q2(R1) together with the accuracy of recovery test for **3** in cilostazol were performed. Also, the linearity of the method was evaluated. The validation of this method was accepted by the involved regulatory agency. The method quantified compound **3** in industrial batches of cilostazol. As a result, the need to change the production method became evident in order to bring the amount of **3** below the established TTC limit of 2.5 ppm. Also, the application of this method to commercially available, formulated cilostazol allowed us to find compound **3** above 2.5 ppm. The application of this HPLC/MS method for compound **3** can help producers of cilostazol to ensure low amounts of this potential genotoxic impurity in the finished API.

## References

[1] ICH Q3B (R2) Impurities in New Drug Products (2006). http://www.ICH.org/.

[2] Raman NVVSS, Prasad AVSS, Ratnakar Reddy K (2011). Strategies for the identification, control and determination of genotoxic impurities in drug substances: A pharmaceutical industry perspective. http://dx.doi.org/10.1016/j.jpba.2010.11.039.

[3] Dow LK, Hansen MM, Pack BW, Page TJ, Baertschi SW (2013). The assessment of impurities for genotoxic potential and subsequent control in drug substance and drug product. J Pharm Sci.

[4] Robinson DI (2010). Control of Genotoxic Impurities in Active Pharmaceutical Ingredients: A Review and Perspective. http://dx.doi.org/10.1021/op900341a.

[5] Elder DP, Lipczynski AM, Teasdale A (2008). Control and analysis of alkyl and benzyl halides and other related reactive organohalides as potential genotoxic impurities in active pharmaceutical ingredients (APIs). J Pharm Biomed Anal.

[6] Müller L, Mautheb RJ, Rileyc CM, Andinod MM, De Antonisd D, Beelse C, DeGeorgef J, De Knaepg AGM, Ellisonf D, Fagerlandh JA, Franki R, Fritschelj B, Gallowayf S, Harpurk E, Humfreyl CDN, Jacksi AS, Jagotam N, Mackinnone J, Mohank G, Nessn DK, O’Donovanl MR, Smitho MD, Vudathalak G, Yottip L (2006). A rationale for determining, testing, and controlling specific impurities in pharmaceuticals that possess potential for genotoxicity. Reg Toxicol Pharmacol.

[7] Bolta HM, Fothb H, Hengstlerc JG, Degena GH (2004). Carcinogenicity categorization of chemicals—new aspects to be considered in a European perspective. Toxicol Lett.

[8] Ashby J (1985). Fundamental structural alerts to potential carcinogenicity or noncarcinogenicity. Environ Mutagen.

[9] Benigni R, Bossa C (2006). Structural alerts of mutagens and carcinigens Curr Comput Aided. Drug Des.

[10] European Medicine Agency (EMeA), Committee For Medicinal Products For Human Use (CHMP) (2006). Guideline on the limits of genotoxic impurities.

[11] ICH guideline M7aon assessment and control of DNA reactive (mutagenic) impurities in pharmaceuticals to limit potential carcinogenic risk (2013). http://www.ema.europa.eu.

[12] USP 37 Monograph / Cilostazol

[13] Reilly MP, Mohler ER (2001). Cilostazol: Treatment of Intermittent Claudication. Ann Pharmacother.

[14] Nishi T, Nakagawa K (1981). Tetrazolylalkoxycarbostyril derivatives and pharmaceutical compositions containing them. US4277479.

[15] Ames BN, Lee FD, Dursto WE (1973). An improved bacterial test system for the detection and classification of mutagens and carcinogens. Proc Natl Acad Sci U S A.

[16] ICH Q2(R1), Validation of analytical procedures: text and methodology Q2(R1) (1996). http://www.ICH.org.

